# Testing the validity of online psychophysical measurement of body image perception

**DOI:** 10.1371/journal.pone.0302747

**Published:** 2024-06-10

**Authors:** Jiří Gumančík, Piers L. Cornelissen, Lise Gulli Brokjøb, Bethany J. Ridley, Kristofor McCarty, Martin J. Tovée, Katri K. Cornelissen

**Affiliations:** 1 Department of Psychology, Northumbria University, Newcastle upon Tyne, United Kingdom; 2 Department of Psychology, The Artic University of Norway, Tromsø, Norway; Union College, UNITED STATES

## Abstract

This body image study tests the viability of transferring a complex psychophysical paradigm from a controlled in-person laboratory task to an online environment. 172 female participants made online judgements about their own body size when viewing images of computer-generated female bodies presented in either in front-view or at 45-degrees in a method of adjustment (MOA) paradigm. The results of these judgements were then compared to the results of two laboratory-based studies (with 96 and 40 female participants respectively) to establish three key findings. Firstly, the results show that the accuracy of online and in-lab estimates of body size are comparable, secondly that the same patterns of visual biases in judgements are shown both in-lab and online, and thirdly online data shows the same view-orientation advantage in accuracy in body size judgements as the laboratory studies. Thus, this study suggests that that online sampling potentially represents a rapid and accurate way of collecting reliable complex behavioural and perceptual data from a more diverse range of participants than is normally sampled in laboratory-based studies. It also offers the potential for designing stratified sampling strategies to construct a truly representative sample of a target population.

## Introduction

Body Image is a complex and multifaceted construct which encompasses an individual’s subjective perspective of their own body, including body-related self-perceptions and self-attitudes that span thoughts, beliefs, feelings, and behaviours [1]. For most individuals, irrespective of gender, body image and appearance are important, as are appearance- and body related concerns [2–4]. A negative body image can contribute to the development of a range of psychiatric and psychological illnesses, including eating disorders and depression [5–8]. By contrast, positive body image is uniquely associated with a sense of well-being, self-care and healthy eating behaviours [9–11].

### Perceptual and attitudinal body image

Consistent with direct measurement [see e.g., 12] and findings from a number of meta-analyses [13–16], a broad distinction can be drawn between perceptual and attitudinal body image. Perceptual body image refers to the accuracy with which a person can judge the physical dimensions of their own body, while attitudinal body image captures the feelings and attitudes that an individual has about their body size and shape.

### Psychophysical measurements of body image

Given the clinical and societal significance of body image, it is important to understand how attitudinal and perceptual body image are measured. Attitudinal body image is usually assessed using a variety of psychometric tasks which examine the four major dimensions of body image: a) overall evaluation of the body in terms of satisfaction, b) affective distress related to appearance, such as stress, anxiety, or discomfort, c) cognitions related to body image, such as appearance schemas, distorted thoughts or beliefs about one’s body, and cognitive investment in appearance, and d) behavioral aspects of body image, including avoidance of situations that evoke concern and body-checking behaviors [17]. The focus of this paper is on psychophysical measures of perceptual body image.

The psychophysical measures of perceptual body image relevant to the current paper are tasks that ask participants to self-estimate their body shape and size, typically indexed by body mass index (BMI). In the last decade such tasks typically utilise photorealistic body shapes made by using computer generated imagery (CGI) techniques to provide the most accurate and ecologically valid stimuli [18–20]. These illustrate photorealistic body shape change as a function of BMI, while retaining the identity of the individual in the stimulus, despite being presented at many different body sizes.

**Fig 1:**
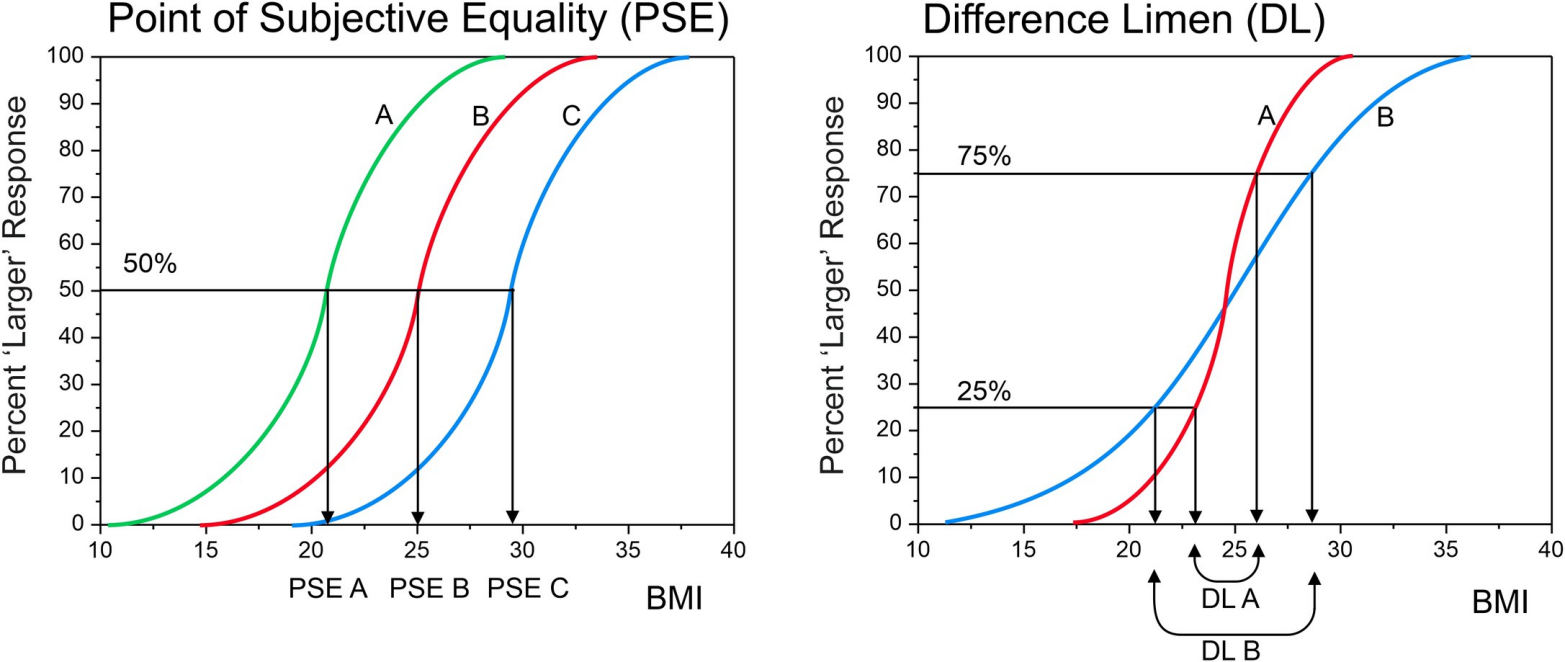
A graphical illustration of how the psychometric function for body size estimation can be used to separate out a participant’s self-estimated BMI (indexed by the point of subjective equality, PSE) from their sensitivity to notice differences in body size (indexed by the just noticeable difference, JND). On the left, participants A, B, and C might all have the same actual BMI of 25. However, participant A under-estimates their body size whilst participant C over-estimates their body size. On the right, participant A is more sensitive to notice change in body size than participant B, and therefore has a steeper psychometric function, with a smaller JND (redrawn from Groves et al., [22]).

Participant’s self-estimated body size can be derived from the responses to such stimuli by using classical psychophysical methods [cf 21]. This is done by using body stimuli to measure two components of participants’ perceptual judgements of body size: (a) the point of subjective equality (PSE) and (b) the just noticeable difference (JND). The PSE is a participant’s estimate of their body size. The JND is an estimate of how sensitive a participant is to changes in body size and equates to the smallest difference in body size that they can detect. The conventional way of obtaining accurate and reliable estimates of the PSE and JND is to use a forced choice task with the method of constant stimuli, but this is only appropriate for a laboratory setting. During each trial of such a task, participants are presented with an image of a CGI body stimulus, and instructed to indicate whether they believe the image presented is bigger or smaller than themselves, by pressing the appropriate button. The BMI of the body shown is randomised throughout the sequence of trials. A psychometric function can then be computed, in which the proportion of ‘larger’ responses (‘stimulus is larger than me’; y-axis) is plotted as a function of the BMI of the stimulus (x-axis), and a sigmoid curve fitted to the data (e.g cumulative Gaussian, Weibul function etc.), as illustrated in Fig 1.

The PSE is then defined from this curve as the BMI at which a participant would report a body stimulus to be larger than them 50% of the time. The midpoint of the PSE curve therefore corresponds with the point (BMI) where the participant would be equally likely to report a stimulus to be ‘smaller’ or ‘larger’ than themselves and can thus be operationalised as the size (BMI) they believe themselves to have. JND (i.e., the smallest BMI change that a participant can notice) is calculated from the BMIs at which a participant would report a body stimulus as larger than herself 25% and 75% of the time (see Fig 1). The “lower” JND is then the difference between the 25% point and the 50% point, while the “upper” JND is the difference between the 75% point and the 50% point. It is common practice to report the average of these two values as the JND [23]. A steep psychometric function signifies high sensitivity to BMI changes, as it indicates that a minor change in BMI significantly influences the participant’s judgment (small JND). Conversely, a flatter curve suggests that participants require more substantial changes in stimulus BMI to notice a difference (large JND). Although, using such a method based on psychophysical principles has been shown to measure participants self-estimated body size reliably and accurately, it has several drawbacks. Primarily, the large number of stimuli presented is both time-consuming and demanding for participants. To use the method of constant stimuli effectively, one must first identify which range of the BMI spectrum is relevant for each individual participant. Then, for accurate estimates, multiple presentations of each stimulus for each BMI are required. For example, for an 11-point psychometric function, stimuli representing 11 small increments of BMI would be required, with each stimulus being presented perhaps 10 times, totalling 110 trials. Therefore, this method demands sustained attention from participants, as they repeatedly perform the same task (deciding if the displayed figure is larger or smaller than their own body). While such focused attention can be achieved in a laboratory setting with researchers monitoring and guiding participant in real time, it is less feasible to expect such reliable performance online, especially with a large number of participants.

As an alternative, we propose the use of the method of adjustment (MoA) as used in the current study. The MoA offers a more efficient approach to measuring perceptual body image, while preserving the foundational psychophysical principles of the more time-consuming method of constant stimuli. As it is much quicker to administer with relatively little loss of precision [23–25], it provides an effective compromise between speed and precision, suitable, in principle, for online studies. In this method, the procedure begins by presenting participants with a body stimulus at a BMI far below or above participant’s self-perceived body size (i.e., PSE). Participants are then asked to use a slider to increase or decrease the body size of the presented stimulus until it aligns with the body size/shape they believe themselves to have. This process is repeated several times, and the participant’s self-estimated body size (PSE) is estimated from the average of these adjustments. The JND is then estimated as the standard deviation of the BMI of this sample of selected body sizes [23]. The MoA’s strength lies in its direct participant engagement and reduced time demand. Participants adjust the stimulus to their perceived body size, eliminating the need for quite so many stimulus presentations as in the method of constant stimuli. The MoA therefore presents a viable option for gaining reliable data in an online setting.

### The merits of body image research in an online setting: Replicability & diversity

A consistent problem for psychology studies has been the unrepresentative nature of participant samples, which have been criticised for a lack of diversity [26–31]. Critiques have pointed out the tendency to draw participants primarily from Western, Educated, Industrialised, Rich, and Democratic (WEIRD) societies. For instance, an analysis of the studies published in three leading psychology journals found that over 90% of the participants were from North America, Europe, Australia, or New Zealand, and less than 3% were from Africa, Asia, Central and South America, and the Middle East [30]. Furthermore, the majority of samples are primarily drawn from specific demographic groups, such as the college students in the United States, often overlooking potential participants from different age groups or those with physical or mental health disabilities [e.g., 26]. This sampling bias has obvious implications for the generalisability of these research findings, as many studies often claim that their findings describe general aspects of human perception or cognition. Without broader and more diverse sampling, it is problematic to extrapolate findings to universal human behaviour.

One practical and efficient solution to the problem of sampling diversity is online testing. The use of paid online services like MTurk or Prolific allows the rapid and cost-effective collection of data from large samples [32, 33]. Beyond its practical advantages, online testing crucially opens the door for more diverse samples from a wider range of unrepresented regions, such as Africa, China, and Latin America. Additionally, these platforms permit precise participant filtering based on specific criteria, giving the option of a more controlled, stratified, sampling strategy. This can result in samples that are representative of a particular population, rather than relying on simple random recruitment and the assumption of representativeness and generalizability. This flexibility from an online design also allows the targeting of specific underrepresented groups, such as ethnic minorities, older or younger people, people of lower socio-economic status, or participants who may have problems in travelling to attend in-person testing, such as people with disabilities. The anonymity that can be provided by online testing might also lead to more honest responses to sensitive questions, as compared to in-person testing [34]. Hence, online testing not only offers a convenient solution but also provides the potential for more representative and honest data.

Despite its numerous advantages, online testing may have certain limitations that can potentially affect data quality [e.g., 35, 36]. One concern is the lack of immediate assistance from a researcher if a participant finds the online instructions unclear, which may make them carry out the study without the necessary care and attention [37]. The latter point is particularly important, as it is difficult to monitor participant engagement in an online environment, which can be a key factor in determining the effect size of the data from the study [38, 39].

### Current study

Given these potential disadvantages, it is crucial to test the viability of running a typical body image study that utilizes the MoA in an online environment. Accordingly, we propose four objectives which, if met, would confirm such viability, and all of which revolve around replicating laboratory-observed phenomena online. Such replication is important to ensure that a particular result is not simply an artifact of experimental conditions or paradigms [40, 41]. This is particularly relevant, as psychology, among other fields, has been grappling with the so-called ‘replication crisis’, a recurring issue pertaining to the inability to replicate the results of a significant number of studies [42–45]. An in-depth replication project focussing on 97 studies from three leading psychology journals reported that only 37% of the results could be replicated [42]. More broadly, the reproducibility of the results reported in published psychology studies seems to fall between 35% and 75%, suggesting a significant proportion of misleading results may distort our attempt to understand human behaviour.

#### Objective 1: Replicability of in-lab estimates of own body size in an online setting

The first objective is to test whether the online replication of self-estimates of body size and shape (indexed by BMI) produces data from the MoA that is equivalent to MoA data that has been gathered in a laboratory setting. This involves using the MoA task online and comparing the results with those collected from an identical task conducted within a controlled laboratory environment. If we can find no difference between the two modes of study, this would suggest that running the MoA online is legitimate.

#### Objective 2: Effects on the PSE: Contraction bias, the influence of attitudinal body image, and the effect of stimulus orientation in an online setting

The second objective was to seek to replicate 4 effects on the PSE that we have observed in the laboratory [see e.g., 18, 46–48]. First, that the slope of the regression of self-estimated BMI (i.e., PSE) on actual BMI has a slope significantly less than 1, where a slope of 1 represents veridical performance (i.e. where self-estimated BMI equals actual BMI). Second, that the cross-over between this regression line and the veridical line occurs at a BMI close to the population mean for the group under consideration. These two effects are evidence of contraction bias [49], whereby actual BMIs less than the population mean are over-estimated, actual BMIs close the population mean are accurately estimated, and actual BMIs above the population mean are under-estimated. Third, attitudinal body image should have a statistically significant and independent effect on self-estimates of BMI, such that, for any given actual BMI, the same increase in body image concerns should give rise to the same fixed increment in self-estimated BMI (i.e., PSE). Fourth, that avatar stimuli oriented in front view should give rise to self-estimates of BMI (i.e., PSE) that are systematically higher than those derived from three-quarter view stimuli, across the full range of actual BMI. Thus, the presence of all four of these effects on the PSE measured in an online environment would provide a credible litmus test for the quality of MoA data collected online.

#### Objective 3: Effects on the JND aligning with Weber’s law in an online setting

The third objective was to test whether data for the JND shows evidence of Weber’s law behaviour. Weber’s law states that the change in the magnitude of a stimulus that will be just noticeable is a constant ratio of the original stimulus magnitude [24]. So, if one compares the size of one object to the size of a reference object, the just noticeable difference (JND) between the two objects (i.e., the minimum size change necessary for an observer to detect a difference) is a constant proportion of the size of the reference object, and this is called the Weber Fraction. For example, if the JND is 5%, then 5% of a small reference object is smaller in absolute terms than 5% of a large reference object. This means that the absolute difference in size required to detect a change in size will be smaller for lower BMI bodies as compared to higher BMI bodies, reflecting their differences in relative size. In practice this means that JND should increase linearly as a function of actual BMI.

#### Objective 4: Test re-test reliability in an online setting

The final objective is straightforward. We wanted to demonstrate test re-test reliability of the MOA measurements obtained from the same participants separated by a minimum of 1 week.

In summary, this study aims to navigate the complexities of transitioning body image research from a controlled laboratory setting to an online environment. It will do so by focusing on three key objectives: a) examining the comparability of online and in-lab self-estimates of body size, b) assessing whether four effects on the PSE that we have observed in the laboratory replicate online, c) assessing whether the JND measured online conforms with Weber’s law behaviour, d) assessing test re-test reliability. Achieving these objectives will provide evidence for the feasibility of online body image research using the MoA task and its potential to yield data consistent with laboratory studies.

## Materials and methods

The study was pre-registered and the data is available to download at osf.io/ektsw.

### Ethics

Ethical approval for this study was granted by the Department of Psychology Ethical Committee at Northumbria University. The first tranche of data was collected between 10^th^ December 2020 and 26^th^ March 2021 (Ethical approval: MyForms ref: 120.3037) and the second tranche of data (the test-retest study) was collected between 14^th^ September and 5^th^ December 2023 (Ethical approval: Project No 4520; Reference: Gumancik 2023-4520-4829). NB–Between when the two studies were run, the ethics system at Northumbria was updated, which is why there is a difference in the format of the ethics codes.

### Sample size

To estimate a suitable sample size for testing objective 2, we used data from Irvine et al., (2019) [50]. This study used a method of adjustment task in a laboratory setting to gather self-estimates of body size from 100 women and measured their actual BMI as well as body satisfaction with the Eating Disorders Examination Questionnaire (EDEQ [51]). An ordinary least squares (OLS) regression model incorporating these two predictors accounted for 65.02% of the variance in self-estimates of body size. The unique variance explained by actual BMI and EDEQ, respectively, was 0.507 and 0.0311. Using this as a template to estimate a sample size for objective 2 in the current study, we employed an OLS multiple regression model with the same predictors. However, we powered the calculation (a fixed model increase in r-square) based on the smaller contribution to the model by EDEQ. This approach estimated that a sample size of 146 women would provide a power of 0.9 at an alpha of 0.05 (G*Power, v3.1.9.6). However, it should be kept in mind that the current study was run online, where it is not possible to validate the accuracy and precision of self-reported height and weight. Further, we expect a high attrition rate because of the multiple number of tasks participants were asked to perform. Therefore, we adopted a highly conservative approach to the final sample size. Using the estimates from the power calculation as a basis, we aimed to gather between 170 and 180 complete datasets from participants who performed all the tasks and to buffer against these uncertainties.

### Participants

All participants were adults over 18 and gave informed consent. As the study was online, the participants first read a briefing document explaining the study and what would be required of them, and then were asked to tick a box if they agreed to take part thereby giving written informed consent.

The final sample consisted of 172 females (Mean age = 26.12, *SD* = 8.42). In this sample, 76.33% referred to themselves as White, 7.10% East Asian, 5.92% as South Asian, 4.73% as Black, and 5.92% as Mixed/Other. When asked about their country of residence, 63.37% of participants responded UK, 9.30% USA, 6.40% Czechia, 4.70% Poland, and 16.86% other countries. In terms of employment status, 52.9% of the sample described themselves as full-time students, 36.6% as full-time or part-time employed, and 9.9% as unemployed. One participant responded “N/A”.

### Psychometric measures

In previous studies [e.g., 18, 46], we have used a rage of psychometric tasks to assess psychological concerns about body shape, weight, and eating, tendency towards depressive symptomatology and self-esteem: i.e., components of attitudinal body image. Typically, this has involved using the Eating Disorders Examination Questionnaire (EDEQ [51]), the Beck Depression Inventory (BDI [52]), the 16-item Body Shape Questionnaire (BSQ-16b [53]), and the Rosenberg Self-Esteem Scale (RSE [54]). Given the fact that we intended to ask participants to carry out the MoA twice, under different instructions, we restricted out psychometric testing to the EDEQ and BDI.

#### Eating Disorders Examination Questionnaire (EDEQ)

The EDEQ is a self-report instrument derived from the Eating Disorders Examination (EDE) interview. The questionnaire is composed of four subscales: (1) the Restraint subscale (EDE-Q res) contains 5 items, which measure the restrictive nature of eating behaviour; (2) the Eating Concern subscale (EDE-Q eat) contains 5 items which measure the preoccupation with food and social eating;(3) the Shape Concern subscale (EDE-Q SC) contains 8 items which measure dissatisfaction with body shape; (4) and the Weight Concern subscale (EDE-Q WC) contains 5 items which measure dissatisfaction with body weight.

Participants report how many days of the past four weeks they have experienced a certain behaviour or thought described in an item, such as “Have you been deliberately trying to limit the amount of food you eat to influence your shape or weight (whether or not you have succeeded)?” Responses are recoded on a 7-point response scale from 0 indicates (no days) to 6 (every day). A global score of overall disordered eating behaviour is also calculated by averaging the four subscales, and frequency data on key behavioural features are recorded. An average global EDE-Q score of 4 is considered the clinical cut-off point. In this this study, the EDE-Q showed excellent internal consistency (*α* = .96).

#### Beck Depression Inventory (BDI)

The BDI is a 21-item questionnaire used to measure levels of depressive symptomatology. The measure presents as a checklist of behavioural and attitudinal items, including “loss of interest,” “sadness,” and “self-dislike.” Each item is rated on a 4-point scale from 0 (no symptom of depression) to 3 (severe expression of a depressive symptom). The total score is a summation of all items ranging from 0-63with higher scores indicating greater levels of depressive feelings. Interpretation of scores are as follows: scores below 10 are considered normal, 11–16 denote mild mood disturbance, 17–20 borderline clinical depression, 21–30 moderate depression, 31–40 severe depression, and scores above 40 indicate extreme depression. For this study, the BDI displayed strong internal consistency with a Cronbach’s alpha of 0.92.

### Stimuli

**Fig 2:**
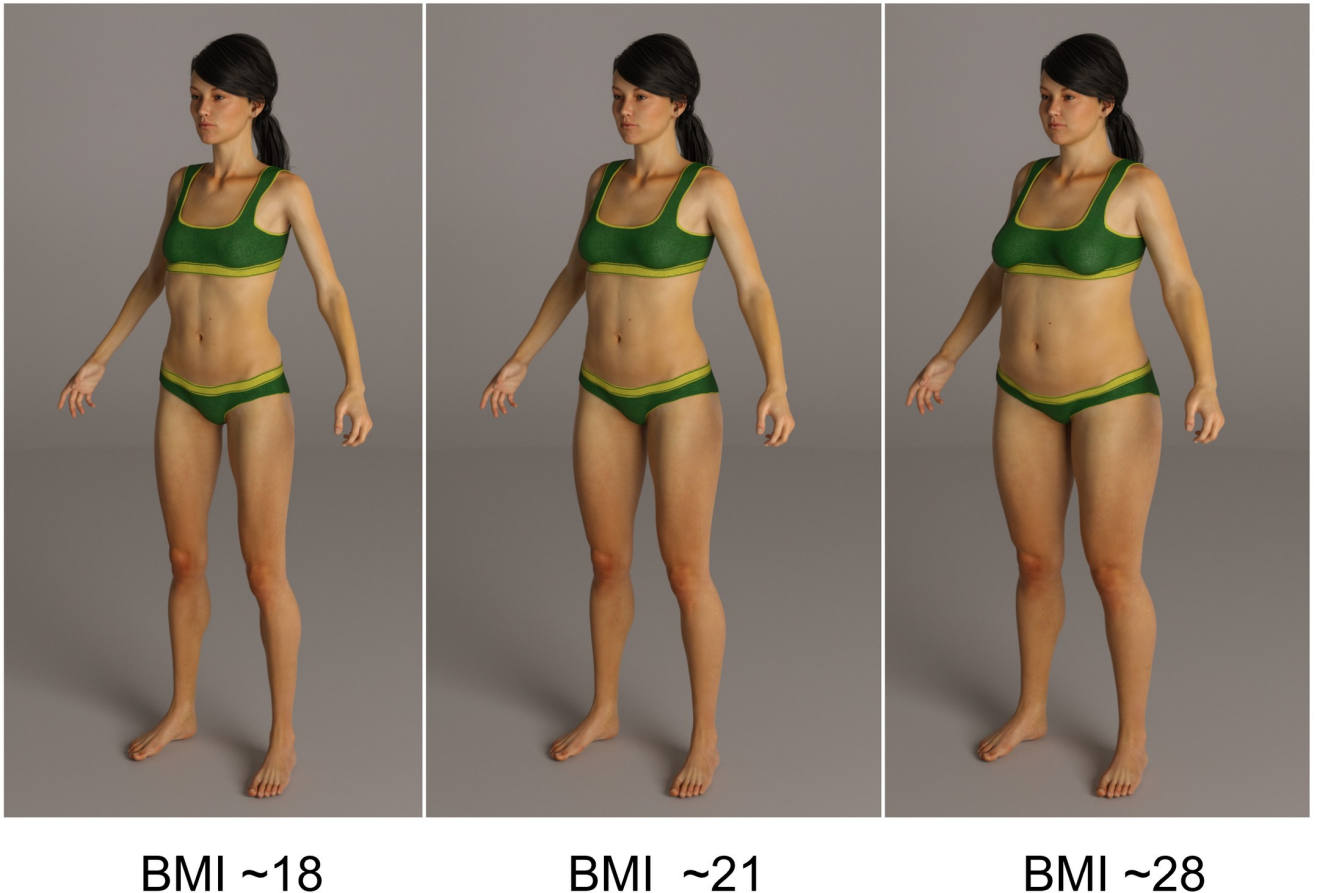
Examples of the CGI images used in the study.

The stimuli used were generated using a 3D Computer-Generated Imagery (CGI) model of an adult female, as illustrated in Fig 2. This model’s dimensions and BMI calibration were based on average data for UK women as obtained from the Health Survey for England [55–57]. By employing DAZ v4.8 software (2017), we generated a sequence of images with a BMI ranging from 12.5 to 44.5 units. Images were rendered using LuxRender software (Version 2.4; Grimaldi et al., 2008 [58]). This approach ensured the stimulus 1) a high level of photo-realistic quality and definition; 2) preservation of the female model appearance across a wide range of BMI values; and 3) demonstrating realistic BMI transitions.

To calculate BMI values for each of the stimulus bodies, we used the Health Survey for England [59–60] datasets to create calibration curves between waist and hip circumferences and height derived from over 5000 women aged between 18 and 45. Because our CGI models exist in an appropriately scaled 3D environment, having set the height of our models to the average height of women in England (1.62 m) we can then measure their waist and hip circumferences, and compare these with our HSE calibration curves to calculate their BMI [61]]. Additionally, we carried out two qualitative checks of the plausibility of the overall body shape changes in our stimuli. Firstly, we compared the shapes of our CGI bodies to a 3D statistical model of the relationship between BMI and shape changes in 114 real bodies [62]. Secondly, we compared our images against a library of digital photographs of 220 women in a standard pose varying from very low BMI (i.e., ∼11 BMI units) to very high BMI (i.e., ∼45 BMI units) [63].

The use of BMI as a de facto proxy for adiposity in this study has some shortcomings. As BMI is based on weight scaled for height, it does not consider the differences in body composition (adipose versus muscle content) that can give rise to the same weight. As a result, the same BMI can describe individuals with a range of body compositions and body shapes [64, 65]. A more anthropometrically accurate approach is to vary body size and shape using multiple variables based on measurements of body composition [19, 66]. However, in this study we have varied body shape based on a single simple parameter (BMI), and it has been argued that at a population level, BMI provides a reasonably good index of body fat changes [67, 68].

### Psychophysical measurement

Psychophysical measurements were taken using The Method of Adjustment (MoA) task, constructed using the PsychoJS JavaScript library part of PsychoPy3 [69]. This task, hosted online by pavlovia.org, needed to be completed using a desktop browser (i.e., not a tablet or mobile phone) and was always presented full screen. To ensure adherence to this, the software was programmed to identify the platform used, and redirected participants accessing the task from a phone or tablet to another page requesting participants to access the task from a desktop or laptop PC.

The same MoA task was used for the two experimental conditions: (a) participants estimating their body size using front view stimuli, and (b) the same participants estimating their body size using three-quarter view stimuli. Each condition comprised 20 trials. Each trial began with the participant clicking a central white plus sign on a black screen. This action triggered the appearance of (a) a task reminder on the left of the screen (i.e., “Find the best match to your own body size/shape”); (b) a stimulus image on the right side of the screen (scaled relatively to 80% of the devices screen height while maintaining the original image aspect ratio); and (c) a white horizontal scale bar (scaled relatively to approximately 33% of the screen width), with a circular red button overlying it at the bottom of the screen. Participants were instructed to interact with the sliding scale to alter the stimulus body size to match their own body size. Dragging the red slider button to the left shrunk the avatar to the lowest BMI (12.5), while dragging it to the right expanded the avatar to the highest BMI (44.5). Once satisfied with the match, participants pressed the space bar, saving the BMI of the selected image for that trial and initiating the next one. The task prohibited participants from moving on without interacting with the slider at least once per trial. Whether the trial started from the maximum or the minimum value was randomised between trials. This was in case there were anchoring effects based on the start point (i.e. if a participant starts at the maximum value on the slider, they will conclude the trial on a higher value than if they started on the minimum value). By randomising the starting position, running 20 trials and averaging the resultant scores we should factor out this bias.

The horizontal location of the stimulus image was jittered horizontally from one trial to the next to prevent participants using spatial cues to remember the location of the red slider button in relation to the stimulus. In addition, the initial appearance of the avatar and the red slider button was randomized between its lowest and highest BMI settings from one trial to the next. The order in which participants carried out the two conditions for the MoA was alternated between successive participants.

### Procedure

Participants were recruited through various channels, including the researcher’s personal contact, social media platforms such as Facebook, Instagram, Twitter, and designated forums for survey exchange such as Surveycircle.com. No financial remuneration was offered to participants. Interested individuals who fulfilled the inclusion criteria were directed to the study via an anonymous link to the Qualtrics survey. Here, the participants were presented with a comprehensive briefing on the nature of the study, the involved paradigms, and the tasks that the study entailed. After providing written informed consent via an online consent form, participants provided demographical information, such as age, gender identity, country of origin, country of current residence, and employment status. They then completed two psychometric questionnaires (EDEQ, BDI), followed by a guide instructing them on accurately measuring their height and weight, illustrated using a gender-neutral avatar. Participants were then presented the psychophysical task, participating in both conditions in the order assigned to them. After finishing the study, participants were informed about the option of withdrawing from the study at any point in case of any psychological or physical discomfort. The study took ∼30 minutes.

## Results

### Univariate statistics

We gathered complete datasets from 172 women. The biometric and psychometric characteristics of the sample are described in Table 1.

**Table 1:** Participant characteristics, n = 172.

	M	SD	Range
**Chronological age (yrs)**	26.12	8.42	18–68
**BMI**	23.02	4.51	15.05–47.47
**BDI**	14.89	10.58	0–48
**EDEQ Global**	2.19	1.6	0–6
**EDEQ Restraint**	2.07	1.89	0–6
**EDEQ Eat**	1.41	1.51	0–6
**EDEQ Shape concern**	2.82	1.8	0–6
**EDEQ Weight concern**	2.47	1.8	0–6

### Multivariate statistics

#### Replicability of in-laboratory estimates of own body size (PSE) in an online setting

The first objective in this study was to evaluate whether the MoA-task used to assess self-estimated body size, produced consistent results across online and laboratory environments. To assess this, we compared the data obtained online from the three-quarter view stimuli in the current study with two previous in-laboratory datasets using the same task and three-quarter view stimuli [12, 50]. Each of these datasets contained data from healthy women, and their body size self-estimates were modelled in relation to actual BMI, age, and performance on several psychometric tasks assessing the attitudinal aspects of participants’ body image.

Table 2 provides a comprehensive summary of the analyses used for comparison. All variables were first centred by converting them to z-scores. Column four lists the explanatory variables initially included in each model, whilst column five indicates the explanatory variables retained after model fitting. Column eight provides the overall model r-square for the final model. Critically, columns six and seven show the beta weight for BMI in each model along with its 95% CI. The close alignment of these beta weights for BMI across all models and the overlapping confidence intervals strongly suggests a negligible influence of study mode, i.e., online versus laboratory, on model outcomes. In other words, the platform of data collection (online vs in-lab) does not significantly impact the reliability of body size self-estimates.

**Table 2:** Re-analysis of 2 laboratory studies and the current online study using three-quarter view stimuli only.

		*N*	Initial IVs	Final IVs	*Β*	95%CI	*R* ^*2*^
**ONL 1**	Current study	172	BMI, Age, EDEQ, BDI	BMI, Age, EDEQ	0.62	0.54–0.70	0.74
**LAB 1**	Cornelissen et al., 2019	40	BMI, Age, BSQ, RSE, EDEQ	BMI, BSQ, RSE	0.60	0.41–0.81	0.77
**LAB 2**	Irvine et al., 2019	96	BMI, Age, BSQ, RSE, BDI	BMI, BSQ	0.68	0.55–0.81	0.67

NB: ONL = Online, LAB = Laboratory

However, from the perspective of traditional ’frequentist’ statistics, these results simply indicate that we are unable to reject the null hypothesis. In this case, the null hypothesis is that there is no difference in self-estimates of body size when studies are conducted online versus in a laboratory. In other words, the frequentist approach, which interprets probability as the long-term frequency of events, has arguably not provided us with enough evidence to (not) claim a significant difference between online and laboratory-based studies.

Therefore, for a more definitive analysis, we also employed a Bayesian analysis of covariance using JASP software [70]. This method enabled us to focus exclusively on the relationship between self-estimated BMI and actual BMI across the 3 studies (Online study 1, lab study 1, lab study 2) side-lining other demographic or psychometric variables for this analysis.

The Bayesian ANCOVA works by comparing four models with varying predictors of estimated BMI: 1) a Null model without predictors; 2) a model containing only study type as a predictor; 3) a model containing only actual BMI as a predictor; 4) a model containing both study type and actual BMI as predictors. Upon analysing the data, only models 3 and 4 showed an increase in their odds after observing data (BFm = 48.380 and BFm = 0.186, respectively). Of these, model 3 emerged as the most probable, P(M|data) = 0.942, making the observed data approximately 16.13 times more likely under this model than model 4. To account for potential model uncertainty, we carried out Bayesian model averaging to test the effects of both predictors [71]. This revealed that the data were ∼10^14^ times more likely under models containing BMI as a predictor, but the likelihood dropped to only 0.062 times when study type was included. Therefore, these analyses suggest that only actual BMI impacts estimated BMI (mean effect = 0.763 estimated BMI units per additional actual BMI unit, 95% credible interval = [0.696, 0.828]), and critically, study environment (online versus laboratory) has no effect.

#### Effects on PSE: Contraction bias, the influence of attitudinal body image, and differential effect of stimulus orientation in an online setting

To begin with Table 3 reports the Pearson correlation matrix between self-estimated BMI (i.e., the equivalent to PSE), JND (i.e., the SD of self-estimated BMI across blocks of twenty trials), participants’ actual BMI, and their psychometric scores on the BDI (index for depression) and EDEQ (index for eating disorder pathology).

**Table 3:** Pearson correlations between outcome and predictor variables.

	Self-estimated BMI	JND	Age	Actual BMI	BDI
**JND**	0.40***				
**Age**	0.44***	0.13			
**Actual BMI**	0.78***	0.26***	0.29***		
**BDI**	0.19**	0.13*	-0.13*	0.11*	
**EDEQ**	0.45***	0.24***	0.11	0.31***	0.54***

NB *** = p < .0001

** = p < .01

* = p < .05

In our second analysis, we sought evidence for four effects on the PSE that have been observed reliably in the laboratory: (a) evidence of contraction bias, defined by a regression slope of PSE on actual BMI slope less than 1; (b) a rotation point of this regression line with respect to the veridical line around the average BMI for women; (c) an independent contribution to estimated body size from participants’ attitudes about their body, indexed by their psychometric performance; (d) self-estimates of BMI derived from stimuli oriented in front view that are systematically higher than those derived from three-quarter view stimuli across the full range of actual BMI (cf. [48, 72–74]). To address these questions, we used PROC MIXED in SAS v9.4 (SAS Institute, North Carolina, USA) to model self-estimated BMI. The starting model included fixed effects for participants’ actual BMI and stimulus orientation (i.e., three-quarter view and front view). Other covariates included participants’ age and their scores on the BDI and EDEQ. We included a random effect to control the intercept for each participant. For dummy coding, front view stimulus orientation was treated as the reference. To compute the denominator degrees of freedom, we specified the Satterthwaite method. Explanatory variables were retained in the final model if the Type III tests of fixed effects: a) were statistically significant at *p* < .05, and b) contributed to a statistically significant reduction in -2 log likelihood. The only exception to this was if one component of a statistically significant interaction term was itself non-significant, it was retained in the model. We explored all possible two-way and three-way interaction terms between fixed effects. The outcomes for the final model of self-estimated BMI (i.e., equivalent to PSE) are shown in the top half of Table 3. In addition, the regression slopes of self-estimated BMI on actual BMI were significantly less than one for both the three-quarter view stimuli avatars (F1, 164 = 248.34, p < .0001) as well as the front view avatars (F1, 165 = 272.05, p < .0001).

**Fig 3:**
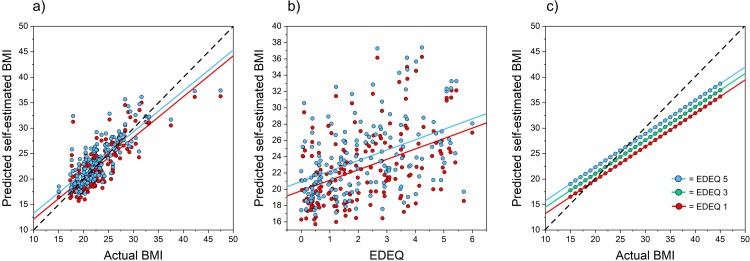
Scatter plots to illustrate the modelled outcomes from top half of Table 3.

The model outcomes described in Table 4 are illustrated in Fig 3.

**Table 4:** Linear mixed effects model for estimated BMI comparing responses from three-quarter and front view avatar presentation.

Outcome	Explanatory Variable	Parameter estimate	*SE*	*t*-value (*DF*)	*p*-value	95% CI
**PSE**	Intercept	3.83	0.99	3.87 (164)	.0002	1.88–5.78
	Three-quarter view	-1.14	0.10	-11.22 (172)	< .001	-1.34 –-0.94
	Front view	0	.	.	.	.
	Actual BMI	0.66	0.044	15.03 (164)	< .001	0.57–0.74
	Chronological age (yrs)	0.13	0.022	5.72 (163)	< .001	0.084–0.17
	EDEQ	0.63	0.12	5.24 (164)	< .001	0.39–0.87
**JND**	Intercept	0.56	0.11	5.12 (169)	< .001	0.34–0.78
	Three-quarter view	-0.11	0.025	-4.34 (171)	< .001	-0.16 –-0.060
	Front view	0	.	.	.	.
	Actual BMI	0.015	0.0048	3.11 (165)	.0022	0.0055–0.025
	EDEQ	0.040	0.014	2.90 (163)	.0042	0.013–0.068

Fig 3A shows the predicted values of self-estimated BMI derived from the model in Table 3, plotted as a function of actual BMI. The blue data points and corresponding regression represent predicted responses to the front view avatar. The red data points and regression line represent predicted responses to the three-quarter view avatar. The dashed black line represents the line of equivalence, where self-estimated BMI exactly matches actual BMI. Two results are apparent in Fig 3A. Firstly, consistent with contraction bias, both regression lines have slopes which are significantly less than 1. Moreover, these two lines display a clockwise rotation relative to the veridical line at an actual BMI of ∼25, close to the population average of UK women. Secondly, self-estimates of BMI for the front view avatars are on average ∼1.1 BMI units higher than for three-quarter view avatars across the full range of actual BMI. Fig 3B shows that for both avatar views (front view and three-quarter view), self-estimated BMI increases systematically as global EDEQ scores increase. In other words, the higher participants’ concerns are about eating, body shape and weight, the higher are their self-estimated BMIs. Notably, this effect is statistically independent of the contraction bias effect. This two-component model for independent contributions from both perceptual and attitudinal body image is illustrated in Fig 3C. The perceptual effect of contraction bias is apparent as a consistent regression slope of < 1, across all three regression lines: actual BMIs below the population average are over-estimated, actual BMIs above the population average (i.e., ∼25) are under-estimated, and actual BMIs close to the population tend to be accurately estimated. Meanwhile, the attitudinal effect, captured by global EDEQ scores, is reflected in the systematic upward shift of the three regression lines as the EDEQ scores increase. This suggests that for any given actual BMI, the self-estimated BMI increases by a fixed amount according to the EDEQ score.

#### JND measures align with Weber’s law in an online setting

In our third analysis, we wanted to test whether performance in the MoA task conformed to Weber’s Law. Specifically, we expected that individual participants’ JND, indexed by the standard deviation of self-estimated BMI across blocks of twenty trials, should increase as a function of participants’ actual BMI. To do this, we used PROC MIXED in SAS v9.4 (SAS Institute, North Carolina, USA) to model the effects of avatar orientation on the standard deviation of self-estimated BMI (i.e., equivalent to JND). We used the same modelling approach and applied the same criteria as for PSE. The bottom half of Table 4 shows the model outcomes for the just noticeable difference.

**Fig 4:**
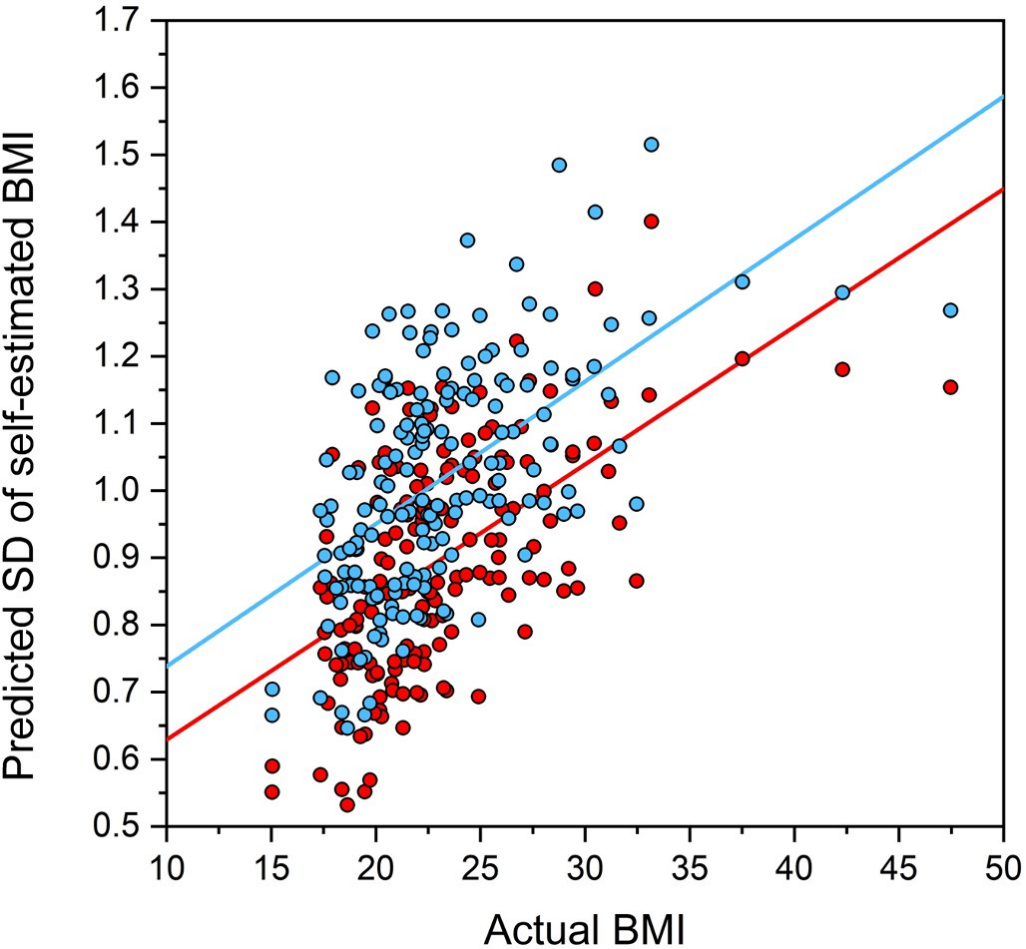
Scatter plots to illustrate the modelled outcomes from bottom half of Table 3.

Fig 4 shows the JND values (indexed by SD of self-estimated BMI) predicted from the model in Table 4 plotted as a function of actual BMI, and separately for the front view avatars (blue data points with blue regression line) and the three-quarter view avatars (red data points with red regression line). Consistent with Weber’s law behaviour, it is clear from Fig 4 that JND increases systematically with actual BMI. Moreover, sensitivity indexed by JND is systematically lower (reflected by higher JND values) for front view avatars than three-quarter view avatars across the full range of actual BMI.

#### Test-retest reliability

As a final step, 36 women (aged 19–52, *M* = 28.37, *SD* = 9.68; BMI 19.69–36.57, *M* = 25.80, *SD* = 4.84) were recruited into a reduced version of the study to explore the test-retest reliability of the MoA task over a 7 to 8-day period. In this sample, 91.43% referred to themselves as White, and 8.57% as South Asian. When asked about their country of residence, 91.43% of participants responded UK, 5.71% Cyprus, and 2.86% other countries. In terms of employment status, 40.00% of the sample described themselves as full-time students, 51.43% as full-time or part-time employed, and 8.57% as unemployed.

On two occasions, separated by at least 7 days but no longer than 14 days, participants were asked to use the MoA to make self-estimates of body size. On each occasion this was done with a block of 20 front facing stimuli, and a second block of 20 three-quarter facing stimuli. Order of presentation of the blocks was randomized. For timepoint 1, participants were first directed to the Qualtrics platform where they provided informed consent before proceeding with the study. Their age, height, weight, ethnicity, country of residence, and employment status were collected. Participants were then directed to Pavlovia.org to complete the new body matrix. Seven days after timepoint 1, participants were contacted with the link to the new body matrix for timepoint 2 and were asked to complete this within seven days. During this session, participants were taken directly to Pavlovia.org to complete the body size judgement task. Each session took approximately 10 minutes to complete. Table 4 shows the mean scores for the test-retest measurements of self-estimated BMI, as well as the correlation between the two time points, which suggest good reliability. To quantify test-retest reliability, recent recommendations suggest using a mixed-effect, two-way model for absolute agreement to compute an intra-class correlation [ICC; 75, 76, 77] Table 5 shows the mean scores for the test-retest measurements of self-estimated BMI, as well as the ICCs between the two time points, which suggest good reliability.

**Table 5:** Mean (SD) scores and intra-class correlation coefficients for self-estimated BMI derived from 3Q and front view stimuli at each timepoint.

	Timepoint 1 *M (SD)*	Timepoint 2 *M (SD)*	ICC	ICC 95% CI
**3Q**	25.39 (6.06)	25.44 (6.12)	0.99	0.98–0.99
**Front**	26.31 (6.08)	26.22 (6.03)	0.98	0.97–0.99

## Discussion

This study aimed to explore the feasibility of conducting a typical body image study online, in which both perceptual and attitudinal body image components are measured. The question at hand was whether the method of adjustment task to obtain self-estimates of BMI would yield the same pattern of results in an online setting as it does in the laboratory. To test this, we established three objectives to determine the feasibility and practicality of running the MoA online.

### Objective 1: Replicability of in-lab estimates of own body size in an online setting

We collected self-estimated BMI data from 172 online study participants using the Method of Adjustment (MoA) task displaying stimuli (CGI avatar) in a three-quarter view. This data was compared with that of 136 participants from two laboratory-based studies who underwent the same MoA task [12, 50]. Specifically, we compared the regression weights in these three studies for actual BMI, derived from multiple regression models in which self-estimated BMI was regressed on actual BMI. These showed very good overlap, indicating no differences between the laboratory versus online mode of study. This overlap implies that the study environment, whether virtual or physical. To enhance the validity of these findings, we further conducted a more formal Bayesian ANCOVA to isolate the influence of actual BMI and the study environment on the self-estimated BMI by using actual BMI as a covariate and the study type as a factor. This analysis provided convincing evidence confirming that only actual BMI impacted self-estimated BMI, and critically, that study environment (online versus laboratory) had no effect. This finding is critical, as it validates that self-estimated BMI, derived from actual BMI measurements, is consistent irrespective of study type (online vs in-lab). In short, this result suggests that laboratory and online self-estimates of BMI, predicted from actual BMI, are equivalent.

### Objective 2: Effects on the PSE: Contraction bias, the influence of attitudinal body image, and the effect of stimulus orientation in an online setting

#### Contraction bias

To further strengthen the validity of online self-estimated BMI measurements using the Method of Adjustment (MoA), we anticipated observing the typical past findings: psychophysical biases—contraction bias and orientation bias. Both tendencies are well-documented phenomena in our laboratory studies, and, as outlined in the Introduction, body size judgements seem to be made by comparison to an internal template distribution based on all the bodies a participant has observed [47–49, 78]. This internal template anchored around a reference BMI, is argued to correspond to the average BMI of the population [49].

One of the key features of this contraction bias is the tendency for body size judgements of bodies close to this reference BMI to be quite accurate, while those considerably deviating from it to be closer to the reference than they are in reality–i.e., there is a contraction of the response range. As a result, the BMI of larger bodies is increasingly underestimated and that of smaller bodies increasingly over-estimated. As is illustrated in Fig 3A and 3C, we did indeed observe these contraction bias effects for self-estimates of BMI using the MoA in both the online and in-lab data. In other words, the contraction bias we have repeated observed in laboratory data was also found in the online data, further indicating the feasibility of using the MoA-task online.

#### Stimulus orientation and body size estimation bias

The second visual bias we wanted to detect in an online setting was bias related to stimulus orientation, which has been consistently observed in laboratory settings. Two such studies have demonstrated that stimuli showing images of bodies in front view give rise to systematically larger estimates of BMI compared to stimuli showing images of bodies in three-quarter view [47, 48]. Consistent with these laboratory studies, the data from the current online study show the same improvement in the accuracy of BMI estimation of stimuli presented in three-quarter view as compared to those in front-view. Notably, this effect held true for both self-estimates of BMI (see Fig 3A) as well as JND (See Fig 4). One reason behind the improved performance when viewing stimuli in a three-quarter view is suggested to be the enhanced visibility of the changes in stomach depth corresponding to variation in BMI [47, 50]. This visual cue is thought to play a key role in BMI judgements across most of the BMI range. However, at the extreme ends of the BMI range, other visual cues, such as the saliency of bony landmarks at very low BMIs, may become important [79, 80]. It is worth noting that this three-quarter view-based advantage may not apply to other body judgements. For example, when judging muscularity, particularly in male bodies, stimuli presented in a front-view may provide more accurate judgments by enhancing the visibility of the V-shaped torso seen in more muscular bodies [66]. Another possible reason for the view-dependent accuracy may lie in the way bodies are judged by the visual system. Prior research in object perception suggests that stimuli are viewed at a 45-degree angle improve recognition and discrimination, referred to as the “canonical” view [81–83]. The theory posits that judgements are made by comparing a given object against a stored reference template, and objects observed from angles like this internal representation are more easily and accurately. Previous studies have suggested that body judgements are made in the same manner [47, 78], pointing to the possibility of a similar canonical advantage in body perception.

#### Independent effect of attitudinal body image

As shown by Fig 3B and 3C, attitudinal body image (indexed here by participants’ EDEQ scores) showed a statistically independent effect on self-estimated BMI. Specifically, for any actual BMI, a given EDEQ score gives rise to the same fixed increase in self-estimated BMI. In other words, our findings suggests that for a given actual BMI, a specific EDEQ score induces the same fixed increase in self-estimated BMI, underlining the significant role of body image attitudes in self-estimations. This, as with the presence of contraction bias in the online data, further validates the use of the MoA-task in an online setting.

### Objective 3: Effects on the JND aligning with Weber’s law in an online setting

Our final objective was to establish whether the online data would replicate the visual bias associated with Weber’s law, a well-known psychophysical principle [24]. Weber’s law states that the smallest detectable difference between a pair of stimuli (the just noticeable difference or JND) maintains a constant proportion, *K*, to the stimulus magnitude. If Weber’s law holds, a plot of the JND for self-estimated BMI (y-axis) as a function of the actual BMI of the participants (x-axis) should be a straight line with a positive slope. This pattern was observed and showed a reasonably consistent Weber fraction: ∼0.03 at a BMI of 15, and ∼0.05 at a BMI of 45. Indeed, the online data displays such a linear relationship, as illustrated in Fig 4. This successful replication of Weber’s law behaviour in an online setting provides further support for the reliability and validity of using the MoA-task in an online setting.

#### Advantages and disadvantages of online testing

A criticism of online testing is the difficulty of verifying participants’ biographical information, including demographic details such as age, gender, profession and SES, as well as anthropometric details such as height, weight, and BMI. Evidence suggests that individuals tend to over-estimate their height and under-estimate their weight [84, 85], with such estimation errors increasing with participant age and BMI size [84, 86]. However, these estimation errors tend to be systematic and can be adjusted by using correction factors [87]. Moreover, online recruitment platforms like Prolific and MTurk, have multiple mechanisms in place designed to filter out participants providing inaccurate biographical data [14].

Another concern is that it has been suggested that online data may be of lower quality than data collected in person [35, 36]. One reason cited is that online participants cannot easily get clarification during the experiment, and may not attend fully to the task, potentially compromising data quality [37, 88]. However, while certain studies have shown some small differences between data from online and in-person collection [89, 90], our study suggests that the quality of data collected online parallels that of our laboratory-based studies.

Online testing does not only provide a fast and efficient of data collection but is also a way of addressing the fundamental issue of sampling representativeness in psychological studies. As has been extensively reported, psychological research often relies on undergraduate volunteers, limiting the generalizability of findings [26–31, 91]. Online recruitment platforms, like Prolific, provide the opportunity to apply a stratified, randomised recruitment strategies using participant recruitment filters. Thus, a genuinely representative population sample can be recruited. Online data collection is also particularly advantageous for reaching under-sampled populations, such as older or disabled populations who might face difficulties and or/reluctance to participate in-person. Online data collection also helps facilitate recruitment from diverse cultures and countries, addressing the common problem of relying on psychology student population, who tend to be Western, Educated, Industrialized, Rich, and Democratic (WEIRD). This is particular concern to the field of body image research, as several studies have suggested that there are notable cross-cultural differences in how body are judged, with significant differences between WEIRD and non-WEIRD populations [92–94]. However, it should be noted that this benefit of such online data collection is, by design, restricted to recruiting participants that can access and navigate the internet. Thus, online recruitment of certain populations is limited to the subsets of that population who can access the internet [95, 96].

## Conclusions

This study demonstrates that a relatively complex behavioural experiment can be conducted online and yield data of the same quality and pattern as their equivalent laboratory data. We propose that online sampling represents a fast and efficient way of collecting reliable behavioural and perceptual data from a wider variety of participants and offers the potential for designing stratified sampling strategies to construct a truly representative sample of the target population.
